# Exploring content and psychometric validity of newly developed assessment tools for itch and skin pain in atopic dermatitis

**DOI:** 10.1186/s41687-019-0128-z

**Published:** 2019-07-16

**Authors:** Louise Newton, Amy M. DeLozier, Philip C. Griffiths, Jennifer N. Hill, Stacie Hudgens, Tara Symonds, Jonathon C. Gable, Jim Paik, Kathleen W. Wyrwich, Lawrence F. Eichenfield, Linda Abetz-Webb, Jonathan I. Silverberg

**Affiliations:** 1 Clinical Outcomes Solutions, Unit 68, Basepoint, Shearway Rd, Shearway Business Park, Folkestone, Kent, CT19 4RH UK; 2 Eli Lilly and Company, Lilly Corporate Center, Indianapolis, IN 46285 USA; 3 Clinical Outcomes Solutions, 53 W Jackson Blvd, Ste 1315, Chicago, IL 6064 USA; 4 Clinical Outcomes Solutions, 1790 E River Rd, Ste 205, Tucson, AZ 85718 USA; 5 Professor of Dermatology and Pediatrics Chief Pediatric and Adolescent Dermatology Vice Chair Department of Pediatrics, University of California San Diego and Rady Children’s Hospital, 8010 Frost St, San Diego, CA 92123 USA; 6 Patient-Centred Outcomes Assessments Ltd, 1 Springbank, Bollington, Macclesfield, Cheshire, SK10 5LQ UK; 7 Dermatology Medical Social Sciences, Preventive Medicine, Northwestern University, NMH/Arkes Family Pavilion, Ste 1600, 676 N Saint Clair, Chicago, IL 60611 USA

**Keywords:** Atopic dermatitis, Itch NRS, Skin pain NRS, Itch, Skin pain, Adolescents, Adults, Health-related quality of life, Patient-reported outcomes, Clinical outcomes assessment

## Abstract

**Background:**

Atopic dermatitis (AD) is a common skin disorder characterized by chronic inflammation, altered skin barrier function, and inflammatory cell skin infiltration that decreases health-related quality of life (HRQoL). The study objective was to understand the patient perspective of AD burden and determine suitable patient-reported outcome (PRO) measures.

**Methods:**

This mixed methods study involved the collection of qualitative and quantitative information from adults (≥ 18 years old) and adolescents (12 – 17 years old) with clinician-confirmed AD regarding their experiences of AD symptoms and its impact on HRQoL. The first part of the study included three stages: in-person concept elicitation (CE) interviews, a 2-week daily electronic diary (eDiary) study, and in-person cognitive debriefing (CD) interviews. An Itch numeric rating scale (NRS) (v1.0) and a Skin Pain NRS (v1.0) evaluation during CD interviews required participants to think about their ‘worst’ itch and ‘worst’ skin pain in the past 24 h. Other PRO measures allowed for psychometric testing. The second part of the study involved telephone-depth interviews (TDIs) and qualitative feedback from participants who had not participated in the CD interviews. Qualitative data were thematically analyzed. Psychometric evaluation of NRS measures was performed using eDiary data.

**Results:**

In the CE interviews, itch and/or itching and skin pain were the most prevalent symptoms consistently discussed by participants. Both NRS measures demonstrated strong psychometric reliability and were applicable across ages with suitable concurrent validity. During the CD interviews, some participants focused their answers on their ‘average’ itch/itching in the past 24 h, rather than their ‘worst’ itch. Some participants answered the Skin Pain NRS thinking about general pain or other types of pain, rather than skin pain specifically. Consequently, modifications to both measures addressed these issues and re-tested as paper-and-pen versions in subsequent TDIs. Itch NRS (v2.0) modifications helped participants focus on their worst itching. Most participants preferred Skin Pain NRS v2.0b, which included skin pain descriptors.

**Conclusions:**

Itching and skin pain are the most important and relevant AD symptoms. The Itch NRS (v2.0) and Skin Pain NRS (v2.0b) appear to be appropriate endpoints for the assessment of itching and skin pain severity for clinical trials with adults and adolescents with AD.

## Background

Atopic dermatitis (AD) is one of the most common, chronic, inflammatory skin diseases [1], with a prevalence of 2% to 10% among adults and 15% to 30% among children in Western geographies [2]. The disease is characterized by widespread skin lesions which can be red, itchy, swollen, cracked, and/or weeping/oozing [3]. Predominant symptoms reported by patients include itch and skin pain [4]. Patients with moderate to severe AD experience a heavy disease burden including physical and psychosocial abnormalities, and a decrease in health-related quality of life (HRQoL), especially in children [5].

The most common measures used to assess AD severity include the Investigator Global Assessments (IGAs) [6], the SCORing Atopic Dermatitis (SCORAD) [7], the Eczema Area and Severity Index (EASI) [8], and the Patient-Oriented Eczema Measure (POEM) [9]. Traditional measures are primarily based on visual assessment of clinical signs and symptoms by physicians. To better understand the burden of AD from the patient perspective, patient-reported outcome (PRO) measures are recommended as an important additional assessment tool [10]. A literature review was conducted which indicated limited evidence of pre-existing PRO tools suitable for use in a clinical trial setting to assess symptoms such as itch and skin pain severity as the main concepts of interest. Of the existing itch measures, the Eppendorf Itch Questionnaire (EIQ), has been reported in three studies in terms of its use and/or measurement properties [11–13], however it has not reported as having been used in the context of a clinical trial. The EIQ is likely unsuitable for clinical trial purposes because it includes 127 items, representing a considerable burden on trial participants. A less burdensome measure, which also has precedence in clinical trial work is the POEM. The POEM is a 7-item HRQoL measure that has been recommended by the HarmoniSing Outcome Measures for Eczema (HOME) group and focuses on the symptoms of AD. However, the POEM assesses each symptom in terms of symptom frequency rather than severity and produces a total score combining the patient’s response to itch, disturbed sleep and dermatological symptoms such as bleeding and flaking skin. Assessing symptom frequency may not reflect the patients’ experience of symptom severity, and a total score consisting of a combination of symptoms may obscure meaningful treatment benefit relating to reduction in specific symptoms. In relation to the measurement of pain in AD, the literature shows that a pain visual analogue scale [14, 15] and the McGill Pain Questionnaire [16], have both previously been used as outcome measures of AD. The measures used in these studies were neither AD specific, nor focused on dermatological-related pain.

The existing tools for the assessment of AD-related itch and skin pain in a clinical trial setting are limited in their ability to accurately assess the impact of new therapies on the patient-reported symptom severity in AD. Per guidance from the Food and Drug Administration (FDA) and European Medicines Agency (EMA), the variability, duration, and frequency of the symptoms measured should be factors in choice of the recall period, and shorter recall periods are typically preferable as reliance on a respondent’s memory over a longer period of time may undermine content validity. Given the high daily symptom variability present in AD, 24-h recall is preferred to allow for the reliable assessment of symptoms [10]. As such, we sought to develop a simple, symptom-specific measures as part of a tool to be administered in a daily diary. Numeric rating scale measures for the daily evaluation of symptoms are commonly used and a low burden way to assess the change in the patients’ condition that have previously been used for the daily assessment of symptomology in clinical trials [17–21].

This study documents the generation of two patient-reported symptom specific measures of AD severity that can be used as clinical trial endpoint measures. This study used a patient-centered, mixed-methods approach to collect and assess patient-reported disease symptoms and impact on HRQoL to gain a better understanding of AD from the patients’ perspective. Specifically, this study aimed to identify the concepts most relevant to participants through concept elicitation, validate the psychometric capabilities of the arising measures and confirm their content through cognitive debriefing with patients.

## Methods

### Study design and participants

**Fig. 1:**
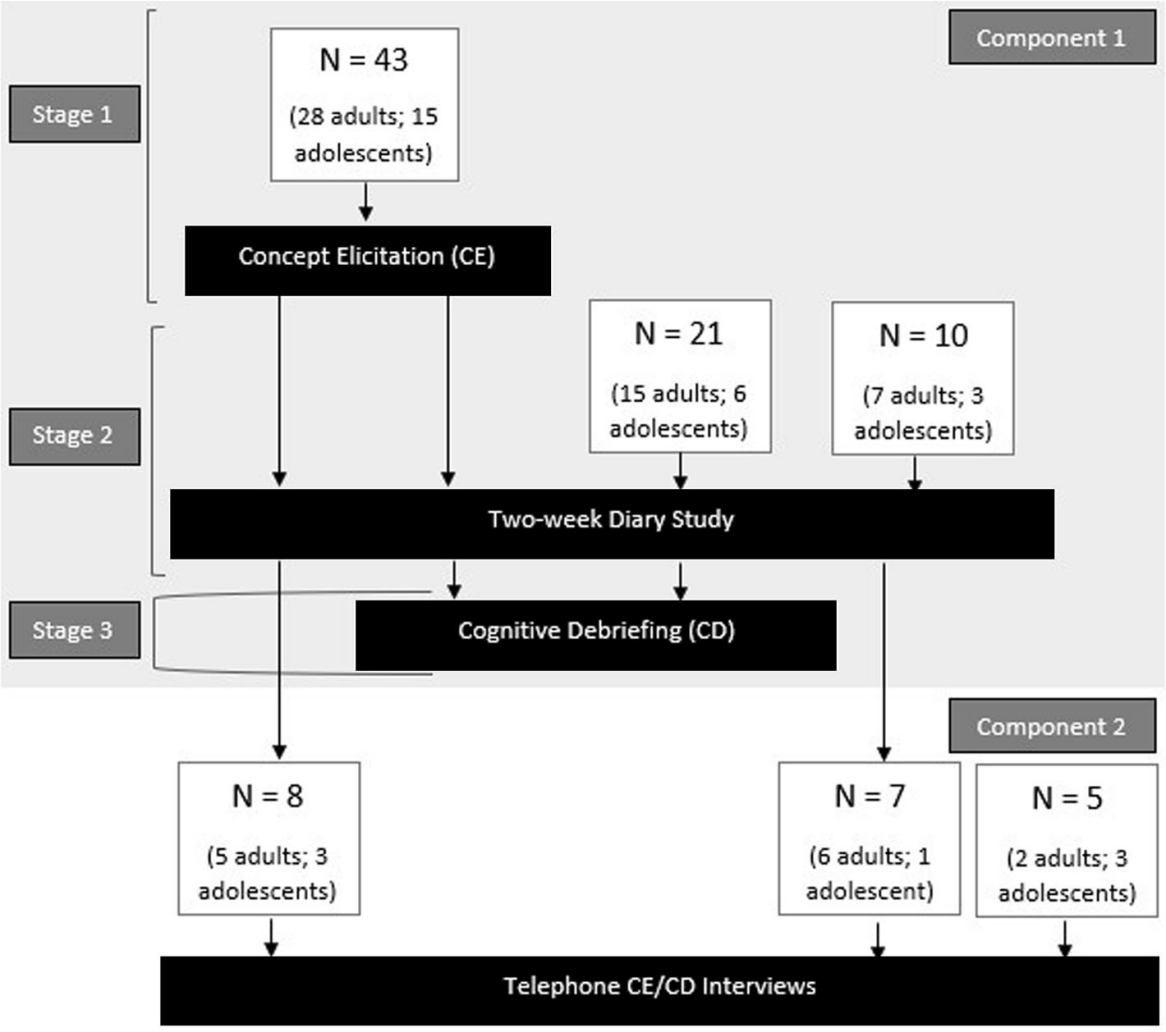
Study Flow

The study was conducted in two components (Fig. 1). Component one involved three stages: in stage one, 43 English-speaking participants from the United States (US) participated in in-person concept elicitation (CE) interviews, including 28 adult participants (≥18 years old) and 15 adolescent participants (12–17 years old) with AD. In stage two of component one, an eDiary study was conducted for 2-weeks involving all 43 participants who had taken part in the CE interviews, as well as another 31 US English speaking participants (22 adults and 9 adolescents) who were newly recruited for this stage. Thus, stage two of component one had a total of 74 participants. In the final stage of component one of the studies, 45 participants (30 adults and 15 adolescents) returned for a cognitive debriefing (CD) interview no more than 2 days after completing stage two. Among the 45 participants, 24 had also taken part in stage one (CE interviews). In component two of the study (which began 16 weeks after component one ended), 20 telephone interviews were conducted to evaluate revisions made to the Itch Numeric Rating Scale (NRS) and Skin Pain NRS. Of the 20 participants (13 adults and 7 adolescents), 13 had also participated in stages one and/or two of component one of the studies.

### Study procedures

Patients aged ≥ 12 years old who were diagnosed with AD for at least 2 years were recruited by a third-party recruitment agency using physician referral from outpatient clinical sites at six cities (Baltimore, Chicago, Los Angeles, St. Louis, New Orleans, and Philadelphia) in the US. Participants were eligible if they had a history of inadequate clinical response to at least one of the following: 1) hydration plus topical steroids and/or antibiotics (eg, tetracycline, trimethoprim, sulfamethoxazole, cephalosporins) and/or topical immune modulators (eg, tacrolimus, pimecrolimus), 2) systemic steroids and/or phototherapy or 3) cyclosporine and/or other immunomodulators (eg, methotrexate, mycophenolate mofetil, azathioprine). Participants were excluded if they had a history of any other clinically relevant skin conditions or a significant psychiatric or physical comorbid condition including abuse of drugs or alcohol in the past 12 months. All participants were able to read, understand and give informed consent and adolescent participants provided assent along with permission/consent from their parent or legal guardian. Additional patient-reported demographic and clinician-reported health data were collected for each participant enrolled by the study recruiter at each site.

The CE interviews were conducted in-person by a trained interviewer using a semi-structured discussion guide. The guide was primarily focused around discussion of participants’ symptom experience with AD and its impact on their HRQoL. The interviewer initially asked open-ended questions designed to elicit spontaneous discussion, which were then followed by in-depth probing around the concepts of interest. Interviews were conducted at a convenient location for the participant and lasted approximately 1 h.

Following the concept elicitation, two 11-point NRS items were developed, each with a 24-h recall period. For itch/itching severity the item required participants to think about their ‘worst itching’ and respond on a scale anchored between 0 = ‘no itch’ and 10 = ‘worst itch imaginable’. For skin pain severity, the item required participants to think about their ‘worst skin pain’ over the previous 24 h and respond on a scale between 0 = ‘no pain’ to 10 = ‘worst pain imaginable’ [22].

For the daily eDiary stage, all participants received training on how to use the daily eDiary. The daily eDiary was programmed to be answered every morning between 5:00 am and 12:00 pm for 14 days. As well as the Itch NRS and Skin Pain NRS, the daily eDiary also contained an adapted version of the POEM which was administered daily and had a 24-h recall period along with two global items: a patient global impression of severity (PGIS) and a patient global impression of change (PGIC). The 7-day recall questionnaires were programmed for completion on Days 0, 7, and 14 of the 2-week period. Participants were sent an email reminder at the start of each day’s completion window. The daily eDiary was programmed so that all questions had to be answered; participants could not submit partial daily eDiary data.

After the 2-week daily eDiary stage, CD interviews were conducted to explore the relevance, appropriateness, and interpretability of the daily eDiary. Per the CE interviews, the CD interviews were conducted in-person by a trained interviewer using a semi-structured discussion guide and lasted approximately 1 h. The CD interviews involved a “think aloud” exercise whereby participants first verbalized their thoughts while completing the Itch NRS and Skin Pain NRS in order to explore their understanding of the items’ instructions, content, recall period and response scale as well as the relevance of the concepts measured. To explore interpretation of score change, participants described what itch and skin pain was like at different points along the response scale of the NRS measures and where on the scale they would be to consider a change in the severity of their itch/itching and skin pain to be meaningful.

For the subsequent telephone CD interviews, the Itch NRS was reworded to “worst itch” to help participants focus on their *worst* itching experience over the previous 24-h. The Skin Pain NRS was also reworded to “worst skin pain” to help patients focus on their *worst* skin pain experience over the previous 24 h. In addition, a second version of the Skin Pain NRS was developed that included descriptors of skin pain in the item anchors to help define the target symptom. Per the in-person CD interviews, the telephone CD interviews focused on the relevance, appropriateness, and interpretability of version 2.0 of the NRS measures and, in addition, participants were asked to select which of the two alternative versions of the Skin Pain NRS (version 2.0a without descriptors or version 2.0b with descriptors) they preferred and why.

### Ethics

All study documents were submitted and approved by the US Copernicus Group Independent Review Board®. The study was performed in accordance with the Declaration of Helsinki and US 21 Code of Federal Regulations [23]. Participants and their referring clinicians received remuneration for their participation.

### Analytic methods

Demographic and health information data were summarized descriptively using data collected at Day 0 for all 74 participants.

Qualitative analyses of CE data were performed using ATLAS.ti (v8) software. Data were coded using a mixed deductive-inductive approach using a pre-specified codebook along with the creation of new codes derived from the data as needed. While describing their experiences of AD, participants were first asked an open-ended general interview question about their experiences, concepts reported in response to this question were coded as *spontaneous.* The interviewer then asked probing questions around the concepts of interest, responses to this question were coded as *probed.* The qualitative analyses of CD data from both the in-person and telephone interviews focused adults’ and adolescents’ understanding and relevance of the target concepts, response scale and options, and recall periods used in the proposed NRS measures.

Measurement properties of the NRS measures were then evaluated using data collected from the eDiary study. This included examination of response distributions (based on eDiary data from Days 1, 7, and 14), test-retest reliability (using intra-class correlation coefficients (ICCs) calculated between Day 7 and Day 14 and between Week 1 and 2 average), concurrent validity (using correlations with the Dermatology Life Quality Index [DLQI], Children’s Dermatology Life Quality Index [CDLQI], Skin Pain NRS, Itch NRS and the PGIS using eDiary data from Days 1, 7, and 14 and the Week 1 and 2 averages (with the exception of the PGIS, no weekly average generated), and responsiveness (based on change categories endorsed on the PGIC scores calculated between Day 7 and Day 14 and between the Week 1 and 2 averages). In terms of guidelines for interpretation, for concurrent validity, the relationship was considered low if the resulting coefficient was < 0.4, moderate if 0.4 – 0.7, and large if > 0.7 [24–26]. For test-retest reliability, ICCs ≥ 0.7 were considered acceptable, ≥ 0.80 were considered acceptable for making judgements on a group basis and ≥ 0.90 for making judgements on an individual participant basis [25]. For responsiveness, significant differences between patients who reported a decline on the PGIC and those who reported stability were supported by effect sizes based on Cohen’s recommendations, the values representing the magnitudes of responsiveness used in this study were considered small if the effect size (ES) = 0.20, moderate if ES = 0.50, and large if ES = 0.80 [26].

## Results

### Study population

The total study population (N = 74) was between 12 and 76 years old with a mean time since AD diagnosis of 11.3 years. The study population included 51.4% female participants and most (41.1%) had moderate AD based on clinician-reported severity (self-reported AD severity was consistent with clinician ratings). Caucasian participants made up 50% of the total study population, followed by African American participants (32.4%). Common comorbidities including asthma, food allergy, and allergic rhinitis were present in approximately 25% of the study population. Conditions such as attention deficit hyperactivity disorder, anxiety, and obesity were present in only a few participants. Table 1 provides a summary of the key characteristics of the study population and by age group.

**Table 1:** Demographic and Clinical Data for the *N* = 74 Total Study Population

	Adolescents (*n* = 24)	Adults (*n* = 50)	Total (*N* = 74)
Age (years)			
Mean (SD)	13.8 (1.94)	48.4 (16.57)	37.2 (21.27)
Median (Min-Max)	13 (12–17)	50 (19–76)	38 (12–76)
Years since diagnosis			
Mean (SD)	6.9 (4.34)	13.3 (17.40)	11.3 (14.86)
Median (min-max)	5 (2–17)	7 (0–47)	6 (0–47)
Clinical severity, n (%)^a^			
Mild	8 (33.3%)	11 (22.4%)	19 (26.0%)
Moderate	10 (41.7%)	20 (40.8%)	30 (41.1%)
Severe	6 (25.0%)	18 (36.7%)	24 (32.9%)
Gender, n (%)			
Female	11 (45.8%)	27 (54.0%)	38 (51.4%)
Male	13 (54.2%)	23 (46.0%)	36 (48.6%)
Race, n (%)			
White/Caucasian	10 (41.7%)	27 (54.0%)	37 (50.0%)
Black/African American	9 (37.5%)	15 (30.0%)	24 (32.4%)
American Indian/Alaska Native	0 (0.0%)	2 (4.0%)	2 (2.7%)
Other/Unknown	5 (20.8%)	6 (12.0%)	11 (14.9%)
Ethnicity, n (%)			
Not Hispanic/Latino	22 (91.7%)	46 (92.0%)	68 (91.9%)
Hispanic/Latino	2 (8.3%)	3 (6.0%)	5 (6.8%)
Unknown	0 (0.0%)	1 (2.0%)	1 (1.4%)
Prior treatment, n (%)			
Yes	23 (95.8%)	43 (86.0%)	66 (89.2%)
No	1 (4.2%)	7 (14.0%)	8 (10.8%)
Comorbidities			
Allergic rhinitis, n (%)	9 (37.5%)	13 (26.0%)	22 (29.7%)
Asthma, n (%)	8 (33.3%)	11 (22.0%)	19 (25.7%)
Food allergy, n (%)	4 (16.7%)	12 (24.0%)	16 (21.6%)
Anxiety, n (%)	0 (0.0%)	3 (6.0%)	3 (4.1%)
Obesity, n (%)	0 (0.0%)	3 (6.0%)	3 (4.1%)
ADHD, n (%)	1 (4.2%)	1 (2.0%)	2 (2.7%)
Patient assessed symptom severity, n (%)			
Not Severe at All	0 (0.0%)	2 (4.0%)	2 (2.7%)
Very Mild	1 (4.2%)	7 (14.0%)	8 (10.8%)
Mild	13 (54.2%)	7 (14.0%)	20 (27.0%)
Moderate	7 (29.2%)	17 (34.0%)	24 (32.4%)
Severe	2 (8.3%)	16 (32.0%)	18 (24.3%)
Very Severe	1 (4.2%)	1 (2.0%)	2 (2.7%)

^a^As assessed by a 5-point Investigator Global Assessment

### Component one: findings from the CE interviews (stage 1)

In the CE interviews, participants (*n* = 43) described experiencing a range of signs and symptoms associated with their AD; the two most frequently reported were skin pain (*n* = 36) and itch (*n* = 35) (Table 2). When participants were asked to name their worst symptom, itch/itching was identified most often by participants (*n* = 17). Itch was described as occurring suddenly and was mainly experienced around the areas of the upper body, including the arms or elbows (*n* = 8), neck (*n* = 6) and chest (*n* = 4). Some locations of itch were more prominent among adults compared with adolescents; for example, adolescents reported itch/itching more frequently in the knee/leg area [33.3% versus 3.6%] and adults reported itch/itching more frequently in the arm/elbow area [21.4% versus 13.3%] and the neck area [17.9% versus 6.7%]. However, all locations were noted at least once in both groups. Some participants described the severity of itch/itching as dependent on its duration while others described severity as the intensity of itch. Seasonality and climate were mentioned as triggers for their itch/itching (*n* = 19, while others described using lotions and creams (*n* = 16), or cold presses and ice packs (*n* = 7) to help relieve their itch. Itch was reported as impacting participants’ lives in multiple ways; *n* = 12 talked about feeling embarrassed because of scratching when itching occurs, and *n* = 10 described how their itch/itching impacts their social life, primarily because of scratching in front of others. Seven participants also reported feeling annoyed, frustrated, and sad due to the frequency with which they experience itch.

Skin pain was characterized as a burning sensation (*n* = 24), soreness or discomfort (*n* = 24), a stinging sensation (*n* = 17), a sensitivity (*n* = 16) and a stabbing feeling (*n* = 8). Some skin pain descriptions were more prominent among adults compared with adolescents; for example, more adults reported burning pain (64% versus 40%), soreness (71% versus 27%), sensitivity (50% versus 13%) and stabbing pain (21% versus 0%), while more adolescents reported stinging pain (67% versus 25%). Participants described their skin pain in terms of their perceptions of severity, which ranged from very minor to very painful/ very severe. Some skin pain descriptors were used to refer to milder sensations; for example, one adult participant rated skin soreness as a 1–4 on a 10-point scale, whereas skin pain she rated as a 5–10. Like itch, the triggers for skin pain included the climate and seasons, contact with the skin, as well as perfumes, scented lotions, and sweat. Applying cold water or a cold towel (*n* = 8) and using creams or lotions (*n* = 7) were mentioned by participants as means of coping with skin pain, however several described the impact it has on their lives including limitations doing physical activities (*n* = 8), at work or school (*n* = 7), and in their social lives (*n* = 6).

**Table 2:** Signs and Symptoms Identified by *N* = 43 Participants in the CE Interviews

Sign/Symptom Identified	Example Participant Quote	Adolescents (*n* = 15)		Adults (*n* = 28)		Total (*N* = 43)
		S	P	S	P	
Skin pain	“I mean, it can be quite severe, actually. I have a pretty high pain threshold … but I’ve had it hurt. I’ve had outbreaks where it was raw and weepy and it hurt enough that I would really cry.” (Male adult)	2	13	3	18	36
Itch	“I have these two spots. One’s on my upper thigh, and one’s on my lower calf. One’s on the right side, one’s on the left side. And they just pop up randomly. They will itch … I think I am going to lose my mind. It’s a bad, bad itch.” (Female adult)	4	8	8	15	35
Redness	“The redness because it makes me look like a baboon’s butt … because it’s super red.” (Male adolescent)	2	13	5	15	35
Scratch/ Scratchy	“It just feels kind of just- very just scratchy.” (Female adolescent)	3	5	8	8	24
Dry skin	“If I was petting … like uh, one of the lizards … rough and … dry.” (Male adolescent)	2	9	8	3	22
Bleeding skin	“It’ll sometimes bleed when I am asleep and then I’ll have blood on my bed and my arm.” Male adolescent)	1	5	3	6	15
Bumps	“They’re like little bumps with like white tips on them. They feel like your … if you have a lot in a place it’ll feel like bumpy.” (Female adolescent)	1	8	1	4	14
Cracking skin	“It almost, like, cracks. I feel like shoe leather, sometimes … you know, real dry and brittle.” (Male adult)	1	4	2	4	11
Rash	“It’s like a rash. Like if you used to have a rash you used to scratch it and be like red-ish and stuff. Just like a whole bunch of bumps in one area.” (Female adolescent)	1	2	4	1	8
Burns	“Then you start to itch, and you feel it … there’s like a little burning feel, like someone’s lighting a match under you … or a lighter.” (Male adult)	1	2	1	2	6
Scaling	“Scaliness is like a snake or something like that … but this is just how the bumps on your skin feel. Like normal. A person who don’t have the condition wouldn’t say a whole bunch of bumps on they skin. They’d say ‘oh you feel like … you feel scaly.’” (Female adolescent)	1	1	2	0	4
Peeling/ flaking skin	“It was just a constant … scratching and flaking and drying skin. It’s improved considerably over the years, but I’ll still get patches where skin will get a dry patch here and a crack.” (Male adult)	0	5	0	2	7
Skin discoloration	“I have it right here on my arm. And they all … usually look like bruises.” (Female adult)	1	0	1	3	5
Inflammation	“Actually, it stays hot the whole time until it is healed. Remember inflammation, part of inflammation is heat.” (Female adult)	0	1	0	4	5
Tightness	“It is almost like a tightness. Like the skin is being pulled tauter.” (Female adult)	0	2	0	2	4
Discomfort	“I don’t know whether the word pain or just discomfort. I think discomfort probably … it’s better than pain because I can’t truthfully say that I’ve been in pain before.” (Male adult)	0	0	0	3	3
Irritation	“Irritation is like when you’re starting a toothache. It’s a little painful when it’s starting. So, if you don’t take care of it, it continues … until it’s time to go to the dentist.” (Female adult)	0	0	0	2	2

*Abbreviations*: *P* Probing, *S* Spontaneously

### Component one: findings from the diary study (stage 2)

**Fig. 2:**
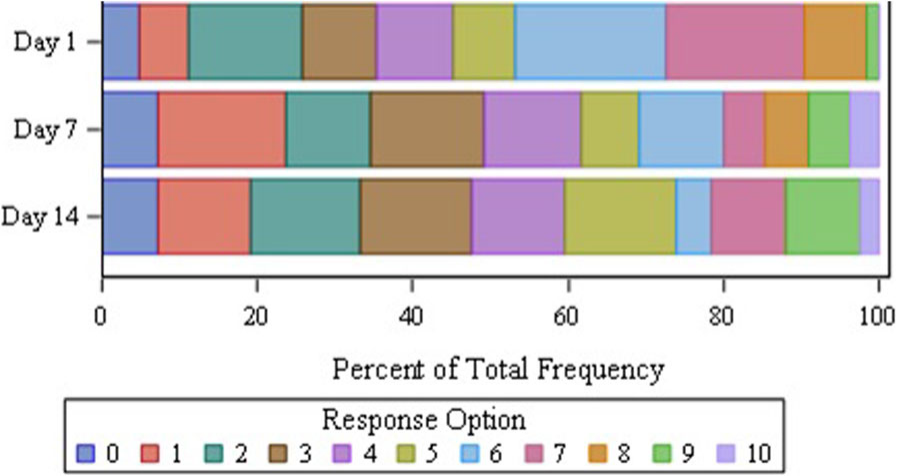
Item Distribution for the Itch NRS and Floor-Ceiling Effects of the Response Levels

The Itch NRS response distribution was examined for floor and ceiling effects on Days 1, 7, and 14 (Fig. 2). Overall, the responses were well distributed, with participants using the majority of the response options at all time points. Floor and ceiling responses were low and ranged from 4.8% to 7.3% and 0% to 3.6%, respectively of the total population. Similar response distributions were observed in the adolescent and adult subgroups. Evaluation of test-retest reliability showed that the Itch NRS was consistent over time (Table 3). For participants who demonstrated no change on the PGIS (*n* = 21), the ICCs between the Day 7 and Day 14 (0.94) and between the Week 1 to Week 2 average values (0.97) were above the ≥0.90 ICC threshold [25]. Psychometric analysis also revealed that the Itch NRS scores showed high correlations with the POEM for both the Week 1 (*r* = 0.80) and Week 2 (*r* = 0.84) average score and moderate to high correlations on Day 1 with the CDLQI summary score (0.76) and the DLQI summary score (0.59) (Table 3). In addition, the Itch NRS had moderate (0.40 ≤ *r* ≤ 0.70) to high correlations (≥ 0.70) with the PGIS at Week 1 (*r* = 0.72) and Week 2 (*r* = 0.74) and on Day 7 (*r* = 0.66) and on Day 14 (*r* = 0.73). The correlations within the adolescent and adult subgroups fluctuated across time points, but generally yielded similar values to the total study population. Overall, participants who reported a decline on the PGIC (*n* = 5) showed a statistically significant greater worsening from the Week 1 to Week 2 average Itch NRS scores than those who did not report a decline (*n* = 24) (mean change of 0.7 versus − 0.4, *p* = 0.020) (Table 3). Participants who showed a decline had a large effect size change (1.21) compared to those who did not show a decline (0.40).

**Fig. 3:**
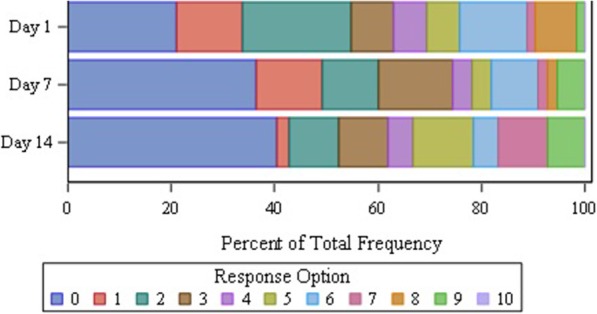
Item Distribution for the Skin Pain NRS and Floor-Ceiling Effects of the Response Levels

Analysis of the response distribution on Days 1, 7, and 14 for the Skin Pain NRS revealed that the most severe response option did not receive endorsement from any participant at any time point (Fig. 3). Although an increase in participants reporting “0” was observed by Day 14, the variability around these numbers due to the low participant counts suggests that this was general variability rather than an improvement over time. Floor responses ranged from 21.0% (on Day 1) to 40.5% (on Day 14) of the total study population. The Skin Pain NRS showed good test-retest reliability with participants who reported no change on the PGIS (*n* = 21) showing consistent scores over time. ICCs between Day 7 and Day 14 (0.91) and between the Week 1 and Week 2 average values (0.97) were above the ≥0.90 ICC threshold. Concurrent validity was also strong with high correlations between the Skin Pain NRS weekly average scores and POEM scores at Week 1 (*r* = 0.75) and Week 2 (*r* = 0.77) and on Day 1 with the CDLQI summary score (*r* = 0.90) and the DLQI summary score (*r* = 0.57). In addition, the Skin Pain NRS had moderate correlations with the PGIS for both Week 1 (0.68) and Week 2 (0.65) average score and on Day 7 (*r* = 0.63) and on Day 14 (*r* = 0.68). The correlations within the adolescent and adult subgroups fluctuated across time points, but generally yielded similar values to the total study population. Analysis of responsiveness revealed that participants who reported a decline on the PGIC (*n* = 5) showed statistically significant greater worsening from the Week 1 to Week 2 average Skin Pain NRS scores than those who did not report a decline (*n* = 39) (mean change of 0.9 versus − 0.3, *p* = 0.003). This is supported by a large effect size of 1.09 in the decline group compared to a small effect size of 0.34 in the group who did not report decline.

**Table 3:** Results of Psychometric Analysis of Real-World Daily eDiary Data

Parameter	Itch NRS^[a]^	Skin Pain NRS^[a]^
Test-retest reliability^[b]^ (*n* = 31)		
Week 1 to Week 2 Average	0.95	0.97
Day 7 to Day 14	0.94	0.91
Concurrent validity^[c]^		
POEM (Week 1; *n* = 74)	0.80	0.75
POEM (Week 2; *n* = 66)	0.84	0.77
PGIS (Week 1; *n* = 55)	0.72	0.68
PGIS (Week 2; *n* = 42)	0.74	0.65
DLQI Summary Score (Adult population; *n* = 33–53)	0.59–0.74	0.57–0.73
CDLQI Summary Score (Adolescent population; *n* = 9–19)	0.76–0.89	0.77–0.91
Responsiveness^[d]^		
Week 1 to Week 2 Average	*p* = 0.02	*p* = 0.003
Decline	ES = 1.21 (*n* = 5)	ES = 1.09 (*n* = 5)
No Decline	ES = 0.41 (*n* = 24)	ES = 0.34 (*n* = 39)
Day 7 to Day 14	*p* = 0.25	*p* = 0.42
Decline	ES = 0.40 (*n* = 5)	ES = 0.24 (*n* = 5)
No Decline	ES = 0.08 (*n* = 24)	ES = 0.04 (*n* = 24)

*Abbreviations*: *CDLQI* Children’s Dermatology Life Quality Index, *DLQI* Dermatology Life Quality Index, *NRS* Numeric rating scale, *PGIS* Patient Global Impression of Severity, *POEM* Patient Oriented Eczema Measure, *ES* Effect size

^[a]^The NRS scales ranged from 0 to 10

^[b]^Test-retest calculation assessed score changes from Day 7 and Day 14 and the Week 1 to Week 2 average in patients who showed a 0- or 1-point change on the PGIS scale

^[c]^Correlations performed on Day 1, 7, 14, and the Week 1 to Week 2 average except for PGIS, which was only collected at Day 7 and 14

^[d]^*p*-value from test for difference in mean score change of participants who scored above and below 4 on the PGIC between Weeks 1 and 2 and Day 7 and Day 14

### Component one: findings from the CD interviews (stage 3)

Forty-five CD interviews were conducted and analyzed to explore participants’ understanding of item content, the relevance of the concept measured, and the appropriateness of the response scale and options as well as the recall period. Overall, there were no differences between adolescent and adult participants in their understanding of the content, relevance, response scale and options, or recall period for the two NRS measures.

A total of 44 participants demonstrated a good understanding of the concept of itch/itching as described in the wording of the Itch NRS. There was clear alignment between the item wording and the language used by participants in the CE interviews to talk about their itch experiences, for example, one adolescent described his experience: *“How do you rate the severity the itch caused by your skin condition over the past seven days? I would um, rate this a little bit higher, it’s moderate, because when my skin got dry because I took a warm bath or I didn’t put on the medicine, then it started getting, then it started itching a little bit, and the longer I didn’t put on the medicine it would start itching more”.* Another adolescent appeared to correctly think about itch/itching as distinct from other symptoms when describing his chosen response to the Itch NRS, however he conflated itch and skin pain when asked to interpret the item using his own words: *“Uh, like how much pain, like how much pain did you have from [inaudible], like zero means, like, none, and 10 means, like, the most you’ve ever felt”*. In addition, participants in the in-person CD interviews commented that the measurement concept of itch/itching was highly relevant based on their own disease experiences, for example, an adult described his experience: *“I itched the day before, and I itched the day before that, and … I’ve been itching the last 20 years”.* Five participants reported that they experience itch but responded ‘0’ to this item because they had avoided triggers. The 0 to 10 response scale was adequate and well understood, for example participants who scored ‘0’ experienced no itch in the past 24 h: “*I didn’t have any itch in the past 24 hours. So, that’s why I selected no itch.”* Indeed, most participants reported thinking back over 24 h when answering the question, supporting the appropriateness of the recall period. When asked, 25 participants appeared to answer the Itch NRS thinking about their itch in general over the past 24 h or how bad their itch/itching was on average compared to their worst itch/itching ever: *“Interviewer: Okay. And so, when I say, ‘worst itching,’ what do you think we mean? Participant: Like, how bad … the intensity.”* Indeed, two participants suggested that the Itch NRS could be improved by providing more explanation of what is meant by ‘worst’ itch: *“a little more details to what’s included in your worst itching, what makes it worse? I was just guessing as far as choosing a number. A six is not as high of a number but higher than average*.”

The Skin Pain NRS was also well-understood by the majority of participants in the in-person CD interviews, noting it as a relevant symptom. Based on qualitative feedback, the response scale and recall period appeared to be suitable for these participants: “*Uh, I’m gonna say a four because it did sting a little last night. Like it did hurt, but it wasn’t too bad. It wasn’t, like, enough, like to make me cry or anything.”* However, there were some participants whose interpretation of the question was not as intended; for example, three participants reported thinking about skin pain and itch/itching when answering the NRS, two participants reported thinking about all types of pain (ie, not specifically skin pain), one participant was unclear about the type of pain being referred to and finally, one participant reported that he excluded skin pain caused by scratching due to itch. Indeed, four participants suggested describing the type of skin pain in the item wording to clarify its meaning. In addition, three participants reported that they had never experienced skin pain due to AD, and one participant felt that other pain descriptors should be used to capture less severe sensations: “*Maybe they could even say, um, irritation, or, like, agitated skin, maybe… It’s just hard to imagine your skin being painful. Like, I just wouldn’t describe it as painful. This [item] is like, I’m a burn victim or something”*. In terms of the level of change participants considered meaningful using the Skin Pain NRS, there was no clear preference, but a 1- or 2-point change appeared beneficial to the majority of participants.

The findings from Stage 2 indicated that the Itch NRS and Skin Pain NRS were psychometrically valid measures of the target concepts and were responsive to changes in condition across the age categories. Both the Itch NRS and the Skin Pain NRS demonstrated reliability over time and exhibited concurrent validity (ie, moderate - high correlations (0.65–0.84) with concurrent measures). Despite these results, the in-person CD interviews conducted in Stage 3 revealed some specific measurement issues based on participants’ feedback. For the Itch NRS, it was not clear if participants were correctly rating their worst itch/itching or averaging across all itch/itching episodes within the 24-h period. In addition, for the Skin Pain NRS, a few participants felt the term “pain” may be too severe or were unclear if milder sensations should be considered when answering this question. Therefore, we made modifications to address these concerns and subsequently tested the revised versions of the NRS measures in telephone CD interviews. The wording of the Itch NRS was revised to help participants appropriately interpret the concept of ‘worst’ itch, ie, “worst level of itching”. To maintain consistency between the two measures, the wording of the Skin Pain NRS was modified in line with these revisions. An alternative version of the Skin Pain NRS was also created with descriptors of skin pain in the item stem with a view to capture pain experiences that participants may consider “less severe” and help participants to focus on skin pain specifically, rather than general pain or other types of pain. The recall period and response scale and options were retained per the original versions of the measures.

### Component two: findings from the telephone CD interviews

In the telephone CD interviews, all 20 participants described their chosen responses to the revised Itch NRS in terms of itch/itching severity or in terms of the intensity of their need to scratch. Of the 13 participants who were asked about their interpretation of the concept of ‘worst itch’ based on how it was described in the item wording, five reported that they answered in terms of how bad their itch/itching had been at its worst point in the day. Eight participants commented that they answered thinking about the severity of their itch/itching over the whole 24-h period and compared this to their worst itch ever. Consistent with the in-person CD interviews, there were no issues raised in terms of the response scale or recall period. Similarly, when asked what score change would be meaningful using the revised Itch NRS, participants asked stated that a 3- to 4-point (*n* = 8; 40%) reduction would be ideal, again consistent with the in-person CD findings. Regarding the two revised versions of the Skin Pain NRS, both were well-understood, yet more participants (*n* = 19 vs. *n* = 14) appeared to understand the target concept when it was presented alongside the skin pain descriptors (v2.0b) compared to the version without the descriptors (v2.0a). For example, two participants were unsure if v2.0a was exclusive to itching/scratching or if it related to skin pain caused by other factors, and four participants interpreted the question as asking about pain *and* itch. In contrast, just one participant mentioned that he thought about itching as well as skin pain when answering v2.0b of the NRS. Participants also gave feedback on the descriptors used in v2.0b. No issues were reported and when asked which other terms could be used to describe skin pain, *n* = 11 participants suggested adding ‘burning/hot’, *n* = 7 suggested ‘ache’ and *n* = 3 suggested ‘stinging’. These terms were similar to those spontaneously used by participants in the CE interviews to describe their skin pain experiences. When asked what score change would be meaningful on each of the two revised versions of the Skin Pain NRS, there was no clear pattern or discernable best score change among participants.

## Discussion

The objective of this research was to incorporate the patient voice in the development of valid and reliable patient outcomes in AD. This study incorporated mixed research methods to ensure that the measurement properties of the two generated measures captured daily symptoms. The first stage of this study was undertaken to gather insights from adults and adolescents regarding the symptoms and impacts of AD from the patient perspective. All participants in the CE interviews commented that itch/itching and skin pain are the worst symptoms associated with AD and described them as priority areas for treatment. Adults and adolescents with AD described very similar disease experiences that negatively affected their quality of life including their ability to do physical tasks, as well as feeling embarrassed or ashamed in front of others. Thus, there was clear overlap between adults’ and adolescents’ AD symptoms and HRQoL experiences, indicating that a consistent measurement approach can be implemented across these age ranges.

Indeed, to capture the severity of itch/itching and skin pain from the perspective of adult and adolescent AD patients, two patient-reported NRS measures were developed and tested in the subsequent stages of this study. In component one, the Itch NRS performed well both qualitatively and quantitatively and was shown to be a valid, reliable, and sensitive measure of AD-related itch. However, revisions were made in response to the finding that not all participants thought about their *worst* itch/itching when answering the question. Findings from the telephone CD interviews (component two) demonstrated that the revisions to the Itch NRS improved participants’ understanding of the target concept consistent with the intended meaning. Similarly, the Skin Pain NRS was shown to be both valid and reliable based on the in-person CD interviews and daily eDiary study, but revisions were also made to maintain consistency with the updated format of the Itch NRS as well as to test the utility of including skin pain descriptors within the item stem itself. Findings from the telephone CD interviews (component two) revealed that both revised versions of the Skin Pain NRS were fit for purpose. However, participants preferred the version with the descriptors as it was better understood and better represented their overall skin pain experiences.

Findings from the quantitative analysis support that original versions of both the Itch NRS and Skin Pain NRS are reliable at both the group and individual patient level [25]. The modifications to the tools were made to improve comprehension rather than to address psychometric weaknesses. Since the modifications were shown to address the comprehension issue, quantitative evidence supporting the original versions will likely hold true, or perhaps be enhanced, for the revised measures. Nonetheless, future research using this revised tool should aim to confirm the psychometric properties of these measures. Specifically, the magnitude of change, denoted by the ES parameter, should be replicated in a larger clinical population. Effect size (ie, ES) is a random variable and sensitive to sample variability which is related inversely to sample size [27, 28]. In this mixed methods study, the observed larger effect size (ES = 0.40) in the stable population can be attributed to the variability in this small sample population.

In the telephone CD interviews, both measures were well understood, but it was noted that over half of participants in component two of the study suggested adding “burning” to the list of descriptors within the Skin Pain NRS (v2.0b). In the CE interviews, a burning sensation was described by some participants as a painful experience but for others, the term was used to describe the redness of their skin or the feeling of heat on their skin that was not seen as a ‘painful’ sensation. Given the different ways the term ‘burning’ was used by participants in the CE interviews, it was agreed that it should not be included as a descriptor of skin pain since those who did not consider it a painful experience would likely find this confusing. Furthermore, the overwhelming majority of participants in the telephone CD interviews understood the concept as it was described in v2.0b of the Skin Pain NRS and felt that the existing descriptors were sufficient in helping them to understand the intended meaning of the concept.

Thresholds for interpreting change on both measures were qualitatively explored; in the CD interviews, both in-person and telephone, participants indicated a meaningful change threshold between 3 and 4 points for the Itch NRS. For the Skin Pain NRS, there was no clear pattern based on qualitative feedback, which may be in part due to participants generally scoring at the low end of the response scale for the Skin Pain NRS. Therefore, further evidence is required to determine the most appropriate responder thresholds for both measures; this is currently being explored as part of a longitudinal, interventional, study with adult AD patients in which full psychometric validation of the revised measures is also being undertaken.

## Conclusions

This study has addressed the previously identified gap in AD PRO measurement literature by creating two measures that assess symptoms of itching and skin pain. Revisions made to improve the validity of the measures were drawn directly from the recommendations of patients with AD who completed and provided feedback on initial versions of the measures and through supplementary interviews with additional AD patients following revisions to the measure. Participants reported day-to-day variations in AD symptomology, and therefore the measures were revised to a daily, 24-h recall period to better capture experience with these symptoms. Existing measures in the field rely on a retrospective 7-day recall period [15, 28], this study suggests that is not adequate for capturing the day-to-day variation experienced by patients with AD and may impact validity of the measure. Further, following the revisions to the measures, this study utilized qualitative and quantitative data from patients with AD show that the new measures are both valid and reliable and adequate for use.

## Data Availability

The datasets generated and/or analyzed during the current study are not publicly available due to individual data privacy, but may be available from the corresponding author on reasonable request. Use of the two measures can be requested from copyrights@lilly.com

## Abbreviations

AD: Atopic dermatitis
CD: Cognitive debriefing
CDLQI: Children’s Dermatology Life Quality Index
CE: Concept elicitation
DLQI: Dermatology Life Quality Index
EASI: Eczema Area and Severity Index
eDiary: Electronic diary
EIQ: Eppendorf Itch Questionnaire
ES: Effect size
HOME: HarmoniSing Outcome Measures for Eczema
HRQoL: Health-related quality of life
ICC: Intra-class correlation coefficients
IGA: Investigator Global Assessment
NRS: Numeric rating scale
PGIC: Patient global impression of change
PGIS: Patient global impression of severity
POEM: Patient-Oriented Eczema Measure
PRO: Patient-reported outcome
SCORAD: SCORing Atopic Dermatitis
TDI: Telephone-depth interview
US: United States

## References

[1] Boguniewicz M, Leung DY (2011). Atopic dermatitis: A disease of altered skin barrier and immune dysregulation. Immunological Reviews.

[2] Okada H (2010). The ‘hygiene hypothesis’ for autoimmune and allergic diseases: An update. Clinical & Experimental Immunology.

[3] Leung DY (2013). New insights into atopic dermatitis: Role of skin barrier and immune dysregulation. Allergology International.

[4] Lewis-Jones S (2006). Quality of life and childhood atopic dermatitis: The misery of living with childhood eczema. International Journal of Clininal Practice.

[5] Lifschitz C (2015). The impact of atopic dermatitis on quality of life. Annals of Nutrition and Metabolism.

[6] Futamura M (2016). A systematic review of investigator global assessment (IGA) in atopic dermatitis (AD) trials: Many options, no standards. Journal of the American Academy of Dermatology.

[7] Kunz B (1997). Clinical validation and guidelines for the SCORAD index: Consensus report of the European task force on atopic dermatitis. Dermatology.

[8] Hanifin J (2001). The eczema area and severity index (EASI): Assessment of reliability in atopic dermatitis. Experimental Dermatology.

[9] Charman CR, Venn AJ, Williams HC (2004). The patient-oriented eczema measure: Development and initial validation of a new tool for measuring atopic eczema severity from the patients’ perspective. Archives of Dermatology.

[10] Food and D (2009). Administration, guidance for industry: patient-reported outcome measures: Use in medical product development to support labeling claims. Federal Register.

[11] Darsow U (2001). New aspects of itch pathophysiology: Component analysis of atopic itch using the ‘Eppendorf itch questionnaire. International Archives of Allergy and Immunology.

[12] Darsow U (1997). Der Eppendorfer Juckreizfragebogen. Der Hautarzt.

[13] Weisshaar E (2012). Questionnaires to assess chronic itch: A consensus paper of the special interest group of the international forum on the study of itch. Acta Dermato-venereologica.

[14] Kawakami T (2006). Oral antihistamine therapy influences plasma tryptase levels in adult atopic dermatitis. Journal of Dermatological Science.

[15] Holm EA (2006). Life quality assessment among patients with atopic eczema. British Journal of Dermatology.

[16] Melzack R (1975). The McGill pain questionnaire: Major properties and scoring methods. Pain.

[17] Ständer S (2013). Pruritus assessment in clinical trials: Consensus recommendations from the international forum for the study of itch (IFSI) special interest group scoring itch in clinical trials. Acta dermato-venereologica.

[18] Dworkin RH (2005). Core outcome measures for chronic pain clinical trials: IMMPACT recommendations. Pain.

[19] Krebs EE, Carey TS, Weinberger M (2007). Accuracy of the pain numeric rating scale as a screening test in primary care. Journal of General Internal Medicine.

[20] Kimball A (2016). Psychometric properties of the itch numeric rating scale in patients with moderate-to-severe plaque psoriasis. British Journal of Dermatology.

[21] Phan NQ (2012). Assessment of pruritus intensity: Prospective study on validity and reliability of the visual analogue scale, numerical rating scale and verbal rating scale in 471 patients with chronic pruritus. Acta Dermato-Venereologica.

[22] Atkinson TM (2010). The brief pain inventory and its “pain at its worst in the last 24 hours” item: Clinical trial endpoint considerations. Pain Medicine.

[23] Association, W.M., World Medical Association Declaration of Helsinki (2001). Ethical principles for medical research involving human subjects. Bulletin of the World Health Organization.

[24] Cronbach LJ (1951). Coefficient alpha and the internal structure of tests. Psychometrika.

[25] Nunnally JC, Bernstein I (1994). Psychometric theory (McGraw-hill series in psychology).

[26] Litwin, M. S., & Fink, A. (1995). *How to measure survey reliability and validity* (Vol. 7). Thousand Oaks: SAGE Publications, Inc.

[27] Hedges LV, Olkin I (1985). Statistical methods for meta-analysis.

[28] Vourc’h-Jourdain M (2009). Patient-oriented SCORAD: A self-assessment score in atopic dermatitis. Dermatology.

